# Cerebrospinal fluid markers of alzheimer’s pathology relate to aMCI among people with HIV

**DOI:** 10.1186/s12883-025-04585-8

**Published:** 2026-01-17

**Authors:** Judith D. Lobo, Vanessa B. Serrano, Laura M. Campbell, Tyler Bell, Ben Gouaux, Douglas Galasko, Scott Letendre, Mark W. Bondi, David J. Moore, Erin. E. Sundermann

**Affiliations:** 1 Department of Psychiatry, University of California, 220 Dickinson St, Suite B, San Diego, CA CA 92103 USA; 2 San Diego Joint Doctoral Program in Clinical Psychology, San Diego, CA USA; 3 San Diego State University/University of California, San Diego, CA USA; 4 Department of Neurosciences, University of California, San Diego, CA USA; 5 Department of Infectious Disease, University of California, San Diego, CA USA

**Keywords:** Amyloid beta, P-Tau, Cerebrospinal fluid, HIV associated neurocognitive disorder, Cognitive decline

## Abstract

**Background:**

As people with HIV survive to older ages, they become more at risk for Alzheimer’s disease (AD) and its precursor, amnestic mild cognitive impairment (aMCI). Memory impairment is also common in other neurocognitive disorders (NDs), including HIV-associated neurocognitive disorders (HAND), which makes it a challenge to diagnose aMCI among PWH. Therefore, we assessed the utility of cerebrospinal fluid (CSF) AD pathology markers in distinguishing aMCI from HAND in PWH by investigating how these markers differentially relate to aMCI.

**Methods:**

Participants included 74 PWH (Mean age = 48 [SD = 8.5]; 87.4% male, 56.5% White) from the National NeuroHIV Tissue Consortium who consented to a lumbar puncture and had CSF biomarker data for Aβ_42_, p-Tau_181_, p-Tau_181_/Aβ_42_ ratio, neopterin, and neurofilament light chain (NfL) data, as well as completed a neurocognitive battery. Participants were classified as aMCI using Jak/Bondi criteria (if they had impairment (≥ 1.0 standard deviation [SD] below the mean) on ≥ 2 memory outcomes among learning, delayed recall, and recognition, with at least one recognition impairment required). HAND participants had impairment (≥ 1.0 SD below the mean) in ≥ 2 cognitive domains. HIV disease characteristics and demographic covariates were included in separate linear regression models that examined how individual biomarkers relate to diagnostic status.

**Results:**

Among participants, 43.2% met the criteria for aMCI, 51.3% for HAND, and 34.2% met the criteria for both HAND and aMCI. Greater AD pathology, specifically CSF p-Tau_181_, was higher in PWH with aMCI compared to PWH without aMCI (F(1,48) = 4.789; *p* =.034; ηp^2^ = 0.091). CSF p-Tau_181_ did not differ by HAND status. While the p-Tau_181_/Aβ_42_ ratio was greater among those with an aMCI-like profile among PWH, it did not reach a statistically significant difference. No other biomarker significantly differed by group status.

**Conclusions:**

Results suggest CSF p-Tau_181_ is indicative of an aMCI-like cognitive profile among PWH, and may be a biologically distinct subgroup of PWH with HAND. More precise diagnosis of aMCI will allow earlier planning; however, more long-term studies are needed to determine the direction of the current results.

## Introduction

Increased effectiveness of antiretroviral therapy (ART) has led to the “graying of the HIV epidemic.” Once a deadly disease, half of people with HIV (PWH) in the USA are over 50 years old, and life expectancy is approaching that of people without HIV (PWoH) [1–3]. However, with the increased survival of older PWH comes risk for age-associated neurodegenerative conditions, including Alzheimer’s disease (AD) [4]. In parallel to their risk for age-associated conditions, HIV-associated neurocognitive disorder (HAND) and other brain health disorders appear to be more common among PWH [5] than PWoH. The neuropsychological profile of HAND in PWH is comparable to that of AD and its common prodromal syndrome, amnestic mild cognitive impairment (aMCI), in that episodic memory impairment, the defining feature of aMCI [6], is a domain commonly impaired in PWH, and individuals with HAND can have episodic memory deficits [7, 8]. Clinical strategies to help distinguish between people who have different cognitive trajectories (healthy aging, aMCI/AD, or HAND) are needed.

Amyloid-β (Aβ) plaque deposits and neurofibrillary tangles of phosphorylated-Tau (p-Tau) protein are hallmark pathological characteristics of AD that develop a decade or more before symptoms [9]. Older adults with aMCI have more advanced CSF biomarkers of AD pathology and are more likely to progress to dementia compared to cognitively normal older adults [10]. Other altered biomarkers in early-stage AD include CSF levels of neurofilament light chain protein (NfL) [11–15], a marker of axonal injury and neurodegeneration, and neopterin, a marker of synaptic damage and neuroinflammation [16–18]. Unlike Aβ_42/40_ and p-Tau_181_, NfL and neopterin alterations are general markers of neurodegeneration and neuroinflammation across neurological diseases [18–20].

Studies of CSF AD biomarkers among PWH are inconsistent. Some studies found that PWH with HAND or cognitive impairment demonstrated biomarker levels reflecting greater Aβ pathology compared to PWH without HAND or cognitive impairment, whereas others found no difference [21, 22]. Multiple studies reported no relationship between p-Tau biomarkers and HAND with cognitive impairment [23–25]; however, a few studies found a relationship between p-tau or the p-Tau/Aβ ratio with HAND or cognitive impairment, although the direction of this relationship differed for the p-Tau biomarkers [24, 25]. Higher levels of NfL predict worse outcomes; another group found that PWH with cognitive impairment had greater NfL concentrations [24, 25]. NfL is associated with elevated levels of neopterin [26]. In a modest sample of PWH with HAND, higher neopterin levels were associated with impaired cognition and alterations in Electroencephalogram (EEG) activity [27]. Among PWH, higher neopterin has been associated with lower hippocampal volume, which was associated with worse episodic memory [13].

Research is needed to differentiate aMCI-associated versus HAND-associated neuropsychological profiles and properly categorize phenotype among PWH to more accurately identify biomarkers that can disentangle aMCI/AD from HAND. Most studies examined AD biomarkers in relation to overall HAND status without examining whether there are also AD-associated cognitive deficits present (e.g., memory). Aβ biomarkers were previously associated with HIV seropositivity, regardless of cognitive status [24, 28–30]. AD pathology may reflect the long-term effects of chronic infection and ART. Our group previously found that higher levels of the p-Tau_181_/Aβ_42_ ratio related to poorer learning/memory performance among PWH but not to cognitive deficits that are typically more specifically associated with HAND, i.e., processing speed and complex visual/motor coordination [31]. It is possible that a combination of p-Tau_181_ and Aβ_42_ measures may be more sensitive to detect an aMCI-like neuropsychological profile among PWH; however, more work is needed to properly classify HAND versus aMCI neuropsychological profiles and examine these classifications in relation to AD biomarkers.

The current study aims to assess the utility of AD pathology markers from CSF to distinguish aMCI from HAND in PWH by investigating how these markers differentially relate to aMCI. We previously adapted the Jak/Bondi aMCI criteria [32] to distinguish HAND from aMCI among PWH. The adaptation was an emphasis on recognition deficits in aMCI diagnosis, which are characteristic of the early AD-associated memory impairment profile but are not common in HAND [33–35]. When applying this adapted criteria to a post-mortem sample of PWH with a cognitive evaluation within a year of death, frontal amyloid pathology was significantly more likely in PWH classified as aMCI versus non-aMCI, whereas the likelihood of frontal amyloid pathology showed no difference by HAND classification [36], suggesting that the adapted aMCI criteria uniquely detect underlying AD pathology. By leveraging our adapted aMCI criteria for PWH, we can more properly characterize clinical phenotype among PWH allowing for a more precise test of the utility of CSF markers of AD pathology and neurodegeneration in disentangling an AD trajectory from HAND. We hypothesized that CSF p-Tau_181_ and the ratio of Aβ_42_/Aβ_40_ levels and p-Tau_181_/Aβ_42_ will relate to aMCI, but not HAND, classification, whereas NfL and neopterin will relate to both aMCI and HAND classification.

## Methods

### Participants

Participants were 74 PWH from the National NeuroHIV Tissue Consortium (NNTC, www.nntc.org) [37] an observational study with few exclusion criteria, with data collected between 2005 and 2016. In the present study, we included those who had available data on CSF Aβ_42,_ p-Tau_181,_ neopterin, and NfL levels, neurocognitive function, and covariates (e.g., HIV duration from the same study visit). The Human Research Protections Program at each NNTC site approved study procedures, and participants provided written informed consent. This study was performed in a manner that was in accordance with the Declaration of Helsinki. The final sample had a mean age of 48 years (standard deviation [SD] = 8.5), within a range of 45 years (minimum age = 25 years old and maximum age = 70 years old). 87.4% were male, and 56.5% were White. All participants completed comprehensive neuromedical, neurocognitive, and neurobehavioral assessments. Clinical trial number: not applicable. CSF biomarkers, neuromedical and neuropsychological evaluations are as described in the previous article [31, 36], however, there was only a partial overlap in study participants and CSF biomarkers used.

### CSF biomarkers

CSF samples were centrifuged at 1200 rpm for 5 min at 4 °C to remove cells, and supernatants were aliquoted and stored at − 80 °C. Our CSF AD biomarkers included levels of p-Tau_181_, the ratio of p-Tau_181_ to Aβ_42_ (p-Tau_181_/Aβ_42_), the ratio of Aβ_42_ to Aβ_40_ (Aβ_42_/Aβ_40_), NfL, and neopterin. Greater pathological burden is reflected by a lower Aβ_40_/Aβ_42_ ratio and higher p-Tau_181_, p-Tau_181_/Aβ_42_ ratio [38], NfL, and neopterin. p-Tau_181_ (Fujirebio, Belgium), Aβ_40_ and Aβ_42_ (Meso Scale Discovery, Rockville, MD), NfL (Tecan, Männedorf, Switzerland) [39], and Neopterin (ALPCO, Salem, New Hampshire) were measured by using commercial immunoassays. The skewed distribution in p-Tau_181_ concentrations was log_10_-transformed. Plates were read on a Meso Sector S 600 imager and data analyzed using MSD Discovery Workbench 4.0 software.

### Neuromedical evaluation

Neuromedical evaluations are as described in a previous article [31]. The estimated duration of HIV disease was self-reported. Nadir CD4 + T-cell count was the lowest lifetime value by either self-report or measurement. CD4 + T-cell count was measured with flow cytometry, and antemortem plasma HIV-1 RNA level was measured by ultra-sensitive PCR (Amplicor, Roche Diagnostic System, Indianapolis, IN; lower limit of detection < 50 copies/ml) in a Clinical Laboratory Improvement Amendments-certified laboratory. Substance use disorder history was assessed by the Composite International Diagnostic Interview [42], and diagnoses were based on the Diagnostic and Statistical Manual of Mental Disorders, fourth edition (DSM-IV).

### Neurocognitive evaluation

A neuropsychological battery assessed seven cognitive domains commonly affected in HAND [40] as previously described, tests included [31]: Hopkins Verbal Learning Test-Revised (HVLT-R) and Brief Visuospatial Memory Test-Revised (BVMT-R)), Wechsler Adult Intelligence Scale-III Digit Span [41], Controlled Oral Word Association Test: FAS Test and Animal naming test, Paced Auditory Serial Addition Test and Letter/Number Sequencing, Trail-Making Test- Part B and Wisconsin Card Sorting Task-64: Preservative Responses and Grooved Pegboard Test. The specific cognitive tests are described elsewhere [42]. Raw test scores were transformed into age-, sex-, education-, and race-adjusted T-scores based on a normative sample of HIV-seronegative participants [43, 44]. The neuropsychological test data were summarized with the Global Deficit Score (GDS) [45], which converts individual T-scores to deficit scores that range from 0 (T-scores > 39) to 5 (T-scores < 20). These individual deficit scores are then averaged to derive the GDS, for which higher scores reflect more significant impairment. GDS values ≥ 0.5 were used to define significant impairment.

*aMCI Classification*. aMCI classification was defined using an adapted version of the Jak/Bondi MCI criteria, which requires two impaired neuropsychological tests (i.e., > 1 SD below demographically corrected normative mean) within a given domain, with this domain being memory for aMCI [32]. In the current study, the learning and recall outcomes included in aMCI classification were the learning, delayed recall, and recognition trials of the HVLT-R and BVMT-R. The adaptation from the original Jak/Bondi MCI criteria was to require that at least one of the two impaired memory outcomes was a recognition test [36].

*HAND Classification.* HAND classification required impairment in GDS, with a cut-point of 0.5 on the total GDS score calculated from neuropsychological tests. Participants who were only impaired in the learning and recall domains were excluded from the HAND group but retained in the aMCI group.

### Statistical analyses

A series of multivariable analyses of covariance (ANCOVAs) was conducted to examine the association between individual CSF biomarker levels (p-Tau_181_, Aβ_42_/Aβ_40_ ratio, p-Tau_181_/Aβ_42_ ratio, NfL, and neopterin) and aMCI or HAND classification, separately, while controlling for covariates. Assumptions were checked for the analysis (i.e. multicollinearity, outliers). Two different models, one with an aMCI status and one with HAND classification, were run for each biomarker variable. Considered covariates included age, sex, race, education, estimated duration of HIV in years, current and nadir CD4 + T-cell counts, ART status, and log of HIV viral load. Covariates were included in the initial models and, if significant at *p* <.10, they were retained in the final model.

## Results

46% of participants were diagnosed with HAND, and 43.2% were diagnosed with aMCI (32.4% with both aMCI and HAND diagnosis). Table 1 displays sample characteristics, including mean AD biomarker levels by aMCI and HAND status.

**Table 1:** Sample characteristics by HAND and aMCI classification

Demographics	HAND	No HAND	*p*-value	aMCI	no aMCI	*p*-value
N (%)	38 (50.7)	37 (49.3)		32 (43.2)	42 (56.8)	
Age (years), mean (SD)	44.9 (10.2)	48.1(9.1)	0.33	46.5(8.7)	45.86(9.9)	0.75
Education, mean (SD)	12.5(2.8)	11.9(2.7)	0.97	12.2(2.7)	12.4(2.8)	0.85
Race n (%)						
White	26(68.4)	19(48.6)	0.03*	18(56.3)	27(64.3)	0.48
Black	11(28.9)	16(43.2)	0.04*	12(37.5)	14(33.3)	0.71
Native Alaskan	1(2.6)	1(2.7)	0.86	1(3.1)	1(2.4)	0.86
Male sex, n (%)	18(47.4)	13(35.1)	0.04*	14(43.8)	13(32.5)	0.009*
HIV Disease Characteristics						
Nadir CD04 + T-cell (cells/ul), mean (SD)	81.6(152)	79.5(149.9)	0.88	89.9(172.4)	75.6(134.7)	0.72
CD4 + T-cell count (cells/ul), mean (SD)	172.1(237)	179.7(209.9)	0.80	185.2(243.9)	159.5(202.9)	0.63
Undetectable Viral Load n (%)	21(63.6)	15(48.5)	0.32	15(55.6)	20(55.6)	0.60
Estimated duration of HIV (yrs) mean (SD)	13.3(5.9)	14.6(6.9)	0.57	13.6(6.3)	13.9(6.3)	0.85
Proportion on ART, n (%)	17(51.5)	23(74.2)	0.79	14(51.9)	26(69.4)	0.80
CSF Biomarkers						
Aβ42 (pg/mL), mean (SD)	2.4(0.2)	2.5(0.3)	0.52	2.4(0.3)	2.4(0.2)	0.74
p-Tau181 level (pg/mL), mean (SD)	1.6(0.2)	1.6(0.2)	0.22	1.6(0.2)	1.5(0.2)	0.06
p-Tau181/Aβ42 ratio, mean (SD)	0.6(0.1)	0.7(0.1)	0.36	0.7(0.1)	0.6(0.1)	0.09
NfL	3.3(0.3)	3.2(0.4)	0.08	3.3(0.4)	3.3(0.3)	0.89
Neopterin	1.3(0.3)	1.3(1.3)	0.34	1.3(0.3)	1.3(0.3)	0.85

32.4% of the participants had a dual diagnosis of aMCI and HAND

*Aβ*_42_ Amyloid Beta 42, *p-Tau*_181_ phosphorylated tau-181, *ART *Antiretroviral medication, *CD4 *Cluster of Differentiation 4 lymphocyte count, *SD *Standard Deviation, *yrs *Years, *NfL *Neurofilament light

### p-Tau_181_concentration

 In an ANCOVA modeling p-Tau181 levels relative to HAND and aMCI classification, covariates included in the final model were age (p = .096) and HIV duration (p = .081) in the aMCI model and age (p = .080) in the HAND model. Results indicated that p-Tau181 levels were significantly higher in those classified as aMCI versus those not classified as aMCI (F(1,48) = 4.789; p =.034; ηp2 = .091), but p-Tau181 levels did not differ by HAND classification (F(1,47) = .061; p = .806; ηp² = .001). 

### Aβ_42_/Aβ_40_Ratio

 In an ANCOVA modeling Aβ42/Aβ40 ratio relative to HAND and aMCI classification, there were no significant covariates in either model. Aβ42/Aβ40 ratio did not significantly differ by aMCI (F(1,72) = .000, p = .987; ηp2 = .001) or HAND classification (F(1, 71) = .388, *p* = .535; ηp2 = .005)*.*

### p-Tau_181_/Aβ_42_Ratio

 In an ANCOVA modeling, the p-Tau181/Aβ42 ratio relative to HAND or aMCI classification, covariates included were age (p = .080) in the HAND model, and there were no significant covariates in the aMCI model. There was a statistical trend towards a significantly higher p-Tau181/Aβ42 ratio level in the aMCI versus no aMCI group (F(1,47) = 3.787, p = .058; ηp2 = .075), whereas levels did not differ by HAND classification (F(1,47) = .061, *p *= .806; ηp2 = .001). 

### Neopterin

 In an ANCOVA modeling neopterin levels relative to HAND or aMCI classification, only HIV RNA (*p* =.015) met the criteria for inclusion in the HAND model, and there were no significant covariates in the aMCI model. Neopterin levels were not significantly associated with aMCI (F(1,18) = 1.231, *p* =.806; ηp^2^ = 0.064) or HAND classification (F(1,18) = 0.220,*p* =.64;5 ηp^2^ = 0.012).

### NfL

**Fig. 1:**
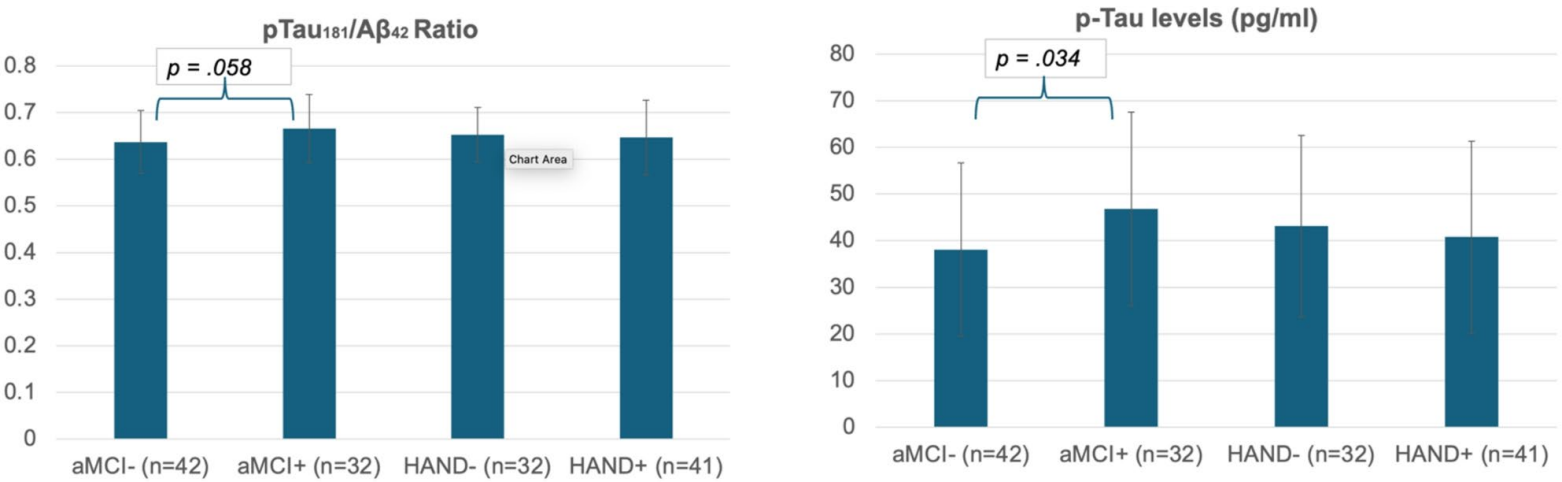
CSF marker Levels by aMCI and HAND groups. Some aMCI + are also in the HAND + group

 In an ANCOVA modeling NfL levels relative to HAND or aMCI classification, age (*p* =.024) met the criteria for inclusion in the HAND model, and there were no significant covariates in the aMCI model. NfL levels were not significantly associated with aMCI (F(1,44) = 0.001, *p* =.976; ηp^2^ = 0.000) or HAND classification (F(1,43) = 1.981, *p* =.166; ηp^2^ = 0.044). Figure 1 displays bar plots of CSF biomarker levels by group.

## Discussion

Our results indicate that the CSF p-Tau_181_ is related specifically to an aMCI-like neuropsychological profile among PWH with high rates of HAND. Thus, p-Tau_181_ may have utility in disentangling aMCI from HAND and informing the need for further diagnostic procedures and intervention. Of note, the p-Tau_181_/Aβ_42_ ratio demonstrated a trend that did not reach statistical significance. This finding aligns with the broader AD literature that shows that tau pathology, rather than Aβ pathology, is more closely tethered to cognitive symptoms [46, 47].

We did not find any group-related differences in two other CSF markers of neurodegeneration and neuroinflammation, NfL and neopterin. This was inconsistent with our hypotheses that NfL and neopterin would be higher among those classified as HAND or aMCI relative to those classified without HAND or aMCI, respectively. While our findings differ from previous work, the samples used in each study are different. Previous studies that compared NfL or neopterin biomarker levels among PWH were from an international sample [27] or using animal models [48]. Further studies are needed to clarify the association between these CSF markers and HAND or aMCI diagnosis.

Consistent with our previous findings, most studies examining biomarkers of p-Tau in relation to HAND among mid-life to older PWH did not find an association, although Cysique et al. (2015) previously found higher CSF p-Tau_181_ levels among PWH with HAND versus without HAND in a study sample with a mean age of 56.7 (SD = 7.9) [21]. Our findings suggest that evidence of a relationship between HAND and p-Tau may be driven by PWH who are experiencing a more AD-like clinical trajectory. It is notable that the Cysique et al. sample was limited to PWH at least 45 years of age, with a third of the sample age sixty and older, and had a somewhat higher percentage of heterozygous (31% vs. 25%) and homozygous [21] (7% vs. 2–3%) carriers of the apolipoprotein E ε4 allele (APOE- ε4) [49], the primary genetic risk factor for sporadic AD. This finding suggests that a relationship between p-tau and HAND may more likely manifest in samples enriched for AD risk factors such as older age and APOE-ε4 positivity.

In a post-mortem study, p-Tau_181_ pathology was associated with neuropsychological performance or memory trajectory over time among PWH with HAND who were younger at age 51 (SD = 0.9) years old [50]. This was in line with findings from our group, where we found that positivity for AD pathology related most consistently to memory-related domains [51]. Our group also previously investigated AD neuropathologic burden from post-mortem brain tissue of PWH in relation to cognitive impairment and aMCI classification. In one study, we found that the presence of Aβ plaques but not p-Tau pathology from the frontal lobe tissue of PWH was more likely in those classified as aMCI versus tissue from other brain regions [36]. In another study in which AD neuropathology was measured from multiple brain regions, the presence of p-Tau pathology in any brain region is specifically related to memory-related domains but only among women with HIV [52]. In that study, a similar pattern was found between greater Aβ pathology in the frontal neocortex and poorer memory scores, although it did not reach a statistically significant difference, while Aβ pathology from other brain regions (i.e., basal temporal neocortex, hippocampus) did not relate to memory scores. This finding suggests that frontal cortex Aβ pathology may be specifically more reflective of an AD-like trajectory in PWH. As CSF Aβ biomarkers are not region-specific but, rather, indices of whole-brain Aβ pathology, this may at least partly explain the discrepancies between our prior, region-specific, postmortem findings and the current finding of a lack of a relationship between CSF Aβ biomarkers and aMCI.

In contrast to AD, the pathogenesis of HAND in the ART era has no clear pathological markers [53]. In post-mortem studies, Aβ_42_ plaque deposits were observed in the post-mortem brains of PWH, particularly in those that had opportunistic infections [54] or HAND [29, 48, 55–60]. However, the Aβ_42_ plaque burden in PWH is typically lower, and the plaque type is more diffuse than in AD patients [5, 48, 56, 59, 61, 62]. In comparison to age-matched, unimpaired persons without HIV, some [29, 30] but not all [63, 64] studies found greater Aβ plaque burden in PWH, regardless of cognitive status. Although less examined, p-Tau pathology is found at autopsy in up to 70% of PWH, with variation between brain regions and as neuropil threads rather than neurofibrillary tangles [50, 51, 65]. When examining p-tau pathology burden in relation to cognition in PWH, one study found no association with cognition [65]. Another study showed that p-Tau burden is specifically related to memory deficits, but not to memory trajectories over time [50, 64]. Finally, another study observed that p-Tau burden related to memory performance solely, but only in women with HIV [51].

A finding that may require further investigation is that the p-Tau_181_/Aβ_42_ ratio had a trending [66, 67] association with aMCI classification and a medium effect size. Our findings may help elucidate the mixed findings in the literature [6, 54, 68, 69] on populations of a similar age range. In a previous study from our group, we examined CSF AD biomarkers in relation to domain-specific cognitive function among PWH. We demonstrated that the p-Tau_181_/Aβ_42_ ratio specifically related to memory measures, similar to the current findings. However, unlike the current findings, p-Tau_181_ alone did not relate to memory measures. This could be due to the limited sample size to detect any differences. There was a 31.08% overlap in the sample between the current study and the prior published study [31].

There was a moderate association between the p-Tau_181_/Aβ_42_ ratio and aMCI status at trending levels. In the general literature, p-Tau biomarkers are more strongly linked to cognitive function than Aβ biomarkers; however, the p-Tau and cognition link tends to be even stronger among those with elevated amyloid markers [70, 71], underscoring the value of a biomarker reflecting both hallmark pathologies. Among older adults without HIV, CSF p-Tau_181_/Aβ_42_ ratios predicted worsening cognitive impairment, both on global cognition and episodic memory in individuals with aMCI and AD [72]. In a study examining older adults with aMCI, individuals with baseline low levels of Aβ_42_/Aβ_40_ and high p-Tau_181_ demonstrated more AD risk and a more dramatic decline across cognitive domains compared to individuals with a diagnosis of vascular dementia [73]. Clifford et al. (2009) further found similar levels of CSF Aβ_42_ and Aβ_40_ among PWH with a HAND diagnosis. In their study, there was an elevation of p-Tau_181_ in PWoH with cognitive impairment compared to PWH with and without HAND [74]. Findings were followed up with a sensitivity analysis to determine if Dementia of Alzheimer’s Type classification was driving the differences between HAND groups by comparing HAND with and without Dementia of Alzheimer’s Type.

The current study has limitations, including the lack of longitudinal data to determine the temporal pattern of results between the cognitive and CSF markers. Longitudinal studies are needed to test whether CSF AD pathology markers are an early marker for incipient cognitive decline and development of aMCI or AD among PWH. Our sample size was limited to those who agreed to lumbar puncture and who had CSF AD biomarker data, which may limit the generalizability of findings. Additionally, the low age of the sample limits the generalizability to older populations at higher risk of AD and may partly explain why associations are weaker or less consistent. It would be informative to compare biomarkers among dual aMCI/HAND classification groups; however, the small cell sizes of the aMCI/HAND groups, particularly the aMCI+/HAND- cell (*n* = 6), precluded us from doing so. Strengths of the study include our empirically based neuropsychological approach to distinguishing aMCI from HAND by exploiting more granular differences in the memory impairment profiles of aMCI/AD and HAND. Ultimately, this leads to more accurate assessments of biomarker associations. Future studies could include other p-tau markers, such as p-Tau_217,_ which has recently been shown to be a particularly sensitive p-Tau biomarker [75].

This study used fluid markers of AD to help identify those who had cognitive impairment more typical of AD, whether or not they also had a diagnosis of HAND. Early detection of aMCI among PWH may be particularly challenging, since PWH exhibit cognitive impairment that spans several cognitive domains despite adherence to ART [76]. Having a more specific diagnosis may help with earlier life planning and inform future interventions aimed at slowing aMCI among those who have comorbid conditions [36].

Overall, these findings suggest that abnormal levels of CSF p-Tau_181_ (and possibly p-Tau_181_/Aβ_42_) are more specific to an aMCI-like neuropsychological profile compared to those diagnosed with HAND. The development of an algorithm or criteria for identifying PWH at high AD risk that involves both clinical and biomarker components would have immense value to clinicians. We see the current findings as a step towards this goal.

## Data Availability

The datasets used and/or analyzed during the current study are available from the corresponding author upon reasonable request.

## Abbreviations

Aβ_42_ and Aβ_40_: Amyloid-β plaque deposits
AD: Alzheimer’s disease
aMCI: Amnestic mild cognitive impairment
APOE- ε4: Apolipoprotein E ε4 Allele
ART: Antiretroviral therapy
BVMT-R: Brief visuospatial memory test-revised
CSF: Cerebrospinal fluid
DSM-IV: Diagnostic and statistical manual of mental disorders, fourth edition
GDS: Global deficit score
HAND: HIV-associated neurocognitive disorders
HIV: Human immunodeficiency virus
HVLT-R: Hopkins verbal learning test-revised
ND: Neurocognitive disorder
NfL: Neurofilament light
NNTC: National neuroAIDS tissue consortium
p-Tau_181_: Phosphorylated-Tau
PWH: People with HIV
PWoH: People without HIV
SD: Standard deviation
